# Proanthocyanidins induce analgesic and anxiolytic effects in spared nerve injured mice by decreasing *in vivo* firing rate of pyramidal cells in the insular cortex

**DOI:** 10.3389/fnmol.2023.1174125

**Published:** 2023-06-23

**Authors:** Pan Wang, Hua-Xing Si, Da-Yu Zhu, Ke-Ke Xing, Jian Wang, Ting-Ting Cao, Han Zhao, Xiao-Die Liu, Ming-Ming Zhang, Tao Chen

**Affiliations:** ^1^Department of Human Anatomy, Histology and Embryology and K.K. Leung Brain Research Centre, The Fourth Military Medical University, Xi’an, China; ^2^College of Life Science, Northwest University, Xi’an, China; ^3^Department of Human Anatomy, Xuzhou Medical University, Xuzhou, Jiangsu, China

**Keywords:** proanthocyanidins, insular cortex, pyramidal cells, neuropathic pain, mouse

## Abstract

Neuropathic pain is one of the most common symptoms of clinical pain that often accompanied by severe emotional changes such as anxiety. However, the treatment for comorbidity of chronic pain and anxiety is limited. Proanthocyanidins (PACs), a group of polyphenols enriched in plants and foods, have been reported to cause pain-alleviating effects. However, whether and how PACs induce analgesic and anxiolytic effects in the central nervous system remain obscure. In the present study, we observed that microinjection of PACs into the insular cortex (IC) inhibited mechanical and spontaneous pain sensitivity and anxiety-like behaviors in mice with spared nerve injury. Meanwhile, PACs application exclusively reduced the FOS expression in the pyramidal cells but not interneurons in the IC. *In vivo* electrophysiological recording of the IC further showed that PACS application inhibited the firing rate of spikes of pyramidal cells of IC in neuropathic pain mice. In summary, PACs induce analgesic and anxiolytic effects by inhibiting the spiking of pyramidal cells of the IC in mice with neuropathic pain, which should provide new evidence of PACs as the potential clinical treatment of chronic pain and anxiety comorbidity.

## 1. Introduction

Neuropathic pain (NP) is a common condition caused by primary disease or lesions damaging the somatosensory pathways from the spinal cord to cortical structures, which has considerable impact on social and economic burden worldwide (Finnerup et al., 2021). The comorbidity of chronic pain and psychiatric disorders such as anxiety is frequently observed in NP patients, and these psychological factors predispose individuals to chronic pain in turn (Zhuo, 2016; Cohen et al., 2021). The process of pain and emotional information is largely regulated by the cortical areas, in which IC is reported to be important (Jasmin et al., 2003; Gamal-Eltrabily et al., 2020; Lee et al., 2022). The IC is activated during harmful stimulation and lesions of the IC decreased pain-related behaviors.

Proanthocyanidins (PACs), a diverse class of oligomeric and polymeric polyphenols, is the main bioactive compound distributed in fruits, seeds, and bark (Anhe et al., 2019). Consumption of PACs has been linked to a range of health benefits by combating aspects of cardiovascular diseases, certain cancers, diabetes, inflammation, and neurodegeneration (Niwano et al., 2022). Besides, several studies report that intragastrical administration and intraperitoneal injection of PACs has analgesic effect underlying molecular mechanisms in suppressing matrix metalloproteinase-9/2 and preserving AKT and ERK activations (Pan et al., 2018; Gao et al., 2020). Recently, our group shows that intrathecal injection of PACs relieves inflammatory pain in the central nervous system at the spinal cord level, and also reveals the synaptic mechanisms and molecular mechanisms of PACs’ analgesic effect (Fan et al., 2021). However, whether PACs relive neuropathic pain and pain-related anxiety has not been investigated at cortical level.

In the present study, we found that microinjection of PACs in the IC alleviated the mechanical allodynia and spontaneous pain and relived anxiety-like behaviors in mice 7 days after spared nerve injury (SNI) surgery. After administration of PACs, the firing rate of pyramidal cells in the IC significantly decreased but the firing rate of interneurons remained unchanged. Our work for the first time shows that PACs contribute to the analgesic and anxiolytic effects by inhibiting the *in vivo* spike of pyramidal cells of the IC in mice with neuropathic pain.

## 2. Materials and methods

### 2.1. Animals

**Figure 1:**
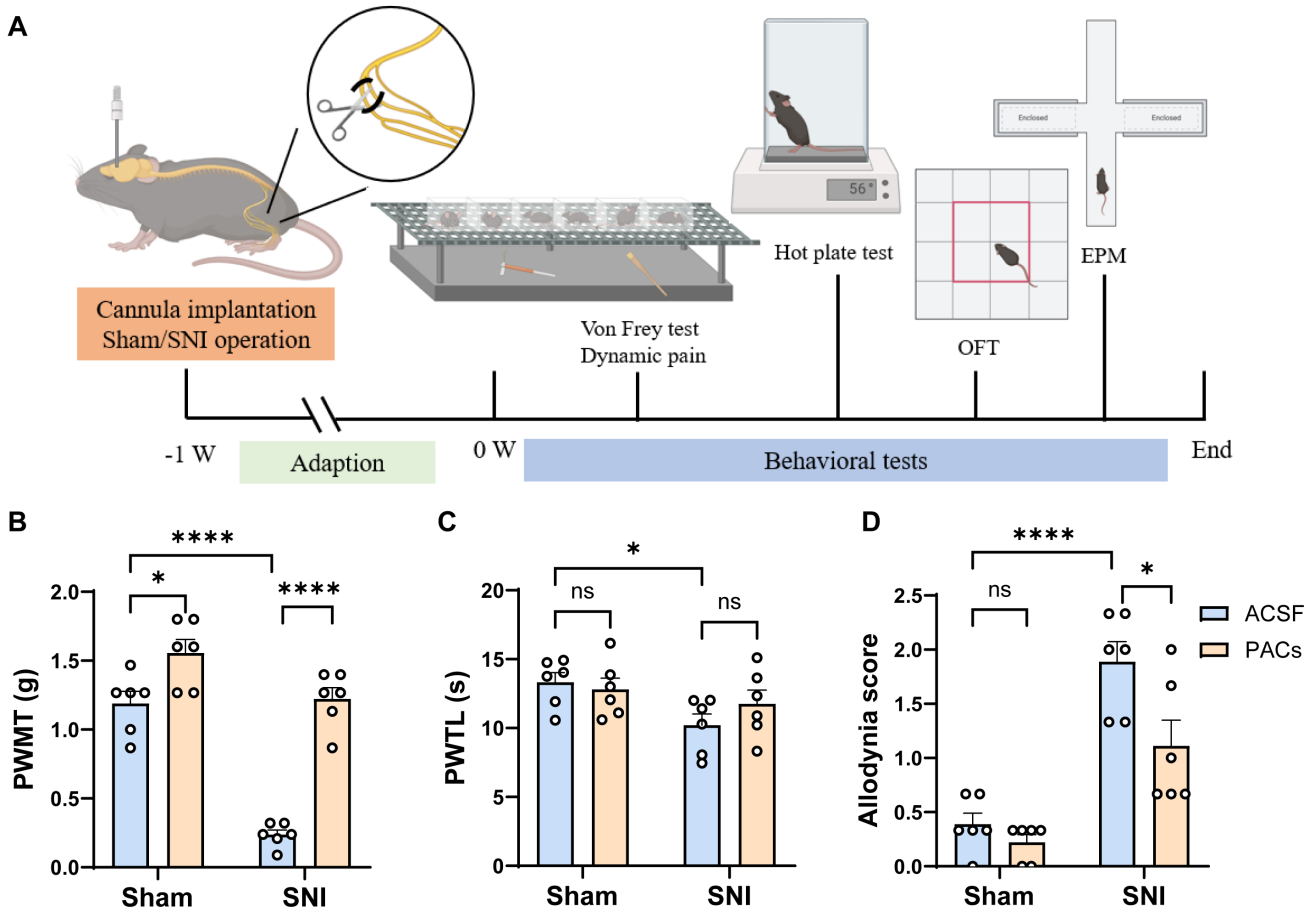
Administration of PACs in IC induces analgesia in SNI mice. **(A)** Experimental timeline. **(B)** The PWMT is significantly decreased in SNI mice in comparison with sham-operated mice (*p* < 0.0001, *t* = 10.02, *n* = 6 mice in each group, unpaired *t*-test). PACs administration significantly increases PWMT when compared with ACSF administration in sham group (*p* = 0.0197, *t* = 2.771, *n* = 6 mice in each group, unpaired *t*-test) and SNI group (*p* < 0.0001, *t* = 11.07, *n* = 6 mice in each group, unpaired *t*-test). **(C)** The PWTL is significantly decreased in SNI mice in comparison with sham-operated mice (*p* = 0.0161, *t* = 2.891, *n* = 6 mice in each group, unpaired *t*-test). PWTL is significant changed neither in sham (*p* = 0.6377, *t* = 0.4856, *n* = 6 mice in each group, unpaired *t*-test) nor in SNI groups (*p* = 0.2598, *t* = 1.195, *n* = 6 mice in each group, unpaired *t*-test). **(D)** PACs significantly reduce the allodynia score in SNI group (*p* = 0.0277, *t* = 2.573, *n* = 6 mice in each group, unpaired *t*-test) but not in sham group (*p* = 0.2094, *t* = 1.342, *n* = 6 mice in each group, unpaired *t*-test). *n.s*., not significant; *p < 0.05; ****p < 0.0001.

The experimental timeline of the study is shown in Figure 1A. Male C57BL/6 J mice (aged 6–8 weeks, weighing 22–26 g) were acquired from the Experimental Animal Center of Fourth Military Medical University. The animals were housed in the laboratory under controlled conditions (temperature: 22 ~ 26°C, humidity: 40%, light/dark cycle: lights on 9 a.m. to 9 p.m.) with food and water available *ad libitum*. After one-week acclimatization, mice were randomly divided into two groups with six mice each. All operations and handling follow the guidelines of the Fourth Military Medical University Ethics Committee.

### 2.2. Drugs

Proanthocyanidins (natural extracts, CAS number: 4852-22-6, Molecular formula: C30H26O13, Molecular weight: 594.52) were purchased from Shanghai Yuanye Biotechnology (Shanghai Yuanye Biological Technology Co., Ltd.). The solution was dissolved in artificial cerebrospinal fluid (ACSF) to produce the original solution at a concentration of 8.41 mM, that is, 5 mg/mL (Fan et al., 2021). The final concentration was diluted in ACSF for behavioral and electrophysiological experiments.

### 2.3. Cannula implantation

Mice were anesthetized with isoflurane (1.0–1.5%) and oxygen (0.6–0.8 liters/min), and then implanted with a 27-gauge stainless steel cannula (RWD, China) with 4.7 mm in length targeted to the IC (1.1 mm anterior to the bregma, 3.0 mm lateral to the midline and 4.7 mm vertical to the skull surface) using a standard stereotaxic apparatus (RWD, China). The cannula was cemented to screws on the skull with dental cement, and a dummy cannula was inserted into the guide cannula. As shown in Figure 1A, the mice were then subjected to Sham/SNI surgery and one-week adaption for recovery. After that, ACSF or PACs were administrated with an injector cannula fixed on a polyethylene (PE50) tube connected to a 5 μL microinjector. The drugs were slowly injected into the IC (0.5 mL/min), and the injection cannula was maintained in place for another 1 min to allow for diffusion.

### 2.4. Neuropathic pain model

The spared nerve injury (SNI) model for neuropathic pain was established as described in our previous study (Zhu et al., 2022). After mice were anesthetized by intraperitoneal injection of 2% pentobarbital sodium (5 mL/kg), three terminal branches of the sciatic nerve were exposed by direct incision of the skin and a section of the biceps femoris muscle in the left thigh. The tibial nerve and the common peroneal nerve were ligated with 6–0 silk sutures and sectioned distal to the ligation. After ligation and cutting, the nerve was put back to its original position and the muscle and skin were sutured in two layers. In the sham operation group, three branches of the sciatic nerve were exposed successively and then disinfected and sutured again, with the nerves not being lesioned.

### 2.5. Pain behavior tests

Measurements were based on previous reports (Zhang et al., 2022). Mice were habituated to the testing environment for 3 days before baseline testing and then placed under inverted plastic boxes (7 × 7 × 10 cm) on an elevated mesh floor and allowed to habituate for 30 min before threshold testing. A logarithmic series of 8 calibrated Semmes-Weinstein monofilaments (von Frey hairs; Stoelting, Kiel, WI, United States) (0.008, 0.02, 0.04, 0.16, 0.4, 0.6, 1, 1.4, and 2 g) with various bending forces (0.078, 0.196, 0.392, 1.568, 3.92, 5.88, 9.8, 13.72, and 19.6 mN) was applied to the plantar surface of the hind paw until the mice withdrew from the stimulus. Positive responses included licking, biting, and sudden withdrawal of the hind paws. A von Frey filament was applied 5 times (3 s for each stimulus) to each tested area. The minimum bending force of the von Frey filament able to evoke 3 occurrences of the paw withdrawal reflex was considered the paw withdrawal threshold. All tests were performed in a blinded manner.

Paw withdrawal thermal latency (PWTL) was measured using the UGO BASILE plantar tenderness instrument (Ugo Basile, Comerio, Italy) as described in previous study (Hargreaves et al., 1988). The mice were put in plastic boxes and adapted to the surrounding environment for 20 min. After that, the radiation light spot of the stimulator was irradiated to the plantar of the mice on the surgical side, and appropriate light stimulation was initiated (the intensity of the beam was adjusted to result in a latency of 8–15 s in Sham mice). Then the latency was measured and recorded every 10 min and repeated for five times. The average value of the results was considered as the threshold of heat pain. In order to avoid tissue damage, the time limit for single irradiation of plantar detection should not exceed 30 s.

The Dynamic mechanical allodynia was tested as described in a previous study (Duan et al., 2014). The mice were placed on an elevated wire grid and covered with a transparent plastic frame. After 30 min of adapting to the surrounding environment in the frame, the plantar hindpaw was stimulated with a paintbrush from heel to toe. During the test, walking away or occasionally raising the foot score 0, raising the foot for more than 2 s or a single gentle retreat score 1, strongly raising the foot above the body level score 2, and continuously shrinking or licking the foot score 3. Each mouse was measured five times with intervals of 3 min, and the average score of the results was calculated.

### 2.6. Anxiety-like behavior

Open field test (OFT). The mice were placed in the OFT system, which was comprised of 8 square chambers (50 cm × 50 cm × 45 cm) (Zhang et al., 2014). Their horizontal movement was detected by a motion tracking system and the central distance and total traveling distance were analyzed by the analysis software (Shanghai Mobile Datum Information Technology, Shanghai, China).

Elevated plus maze (EPM). The following day, the mice were subjected to EPM test system consisted of two open arms (30 cm × 5 cm) and two closed arms (30 cm × 5 cm × 15 cm) as described in previous studies (Wang et al., 2015, 2021). Their horizontal movement was detected by a motion tracking system and the central distance and total traveling distance were analyzed by the analysis software (Shanghai Mobile Datum Information Technology, Shanghai, China).

### 2.7. Immunofluorescent histochemical staining

Immunofluorescent histochemical procedure was applied to evaluate the double-labeling of FOS/CaMKII and FOS/GAD67 in the IC of sham and SNI mice as described in our previous study (Zhang et al., 2022; Zhu et al., 2022). Briefly, mice were perfused with 0.1 mol/L PBS and 4% paraformaldehyde for fixation, and then serially cut into transverse slices with 30 μm thickness. All serial sections were then incubated with primary antisera (1:400, ab11959, Abcam, MA, United Kingdom) for 18–24 h at 4°C in 0.01 M PBS containing 1% (v/v) normal donkey serum, 0.3% (v/v) Triton X-100, 0.02% (w/v) sodium azide, and 0.12% (w/v) carrageenan (pH 7.4). Then, the sections were incubated with Alexa 488 donkey anti-rabbit (1:500, A21206, Invitrogen)/Alexa 594 donkey anti-mouse (1:500, A21203, Invitrogen, CA), and Alexa 488 donkey anti-mouse (1:500, A21202, Invitrogen)/Alexa 594 donkey anti-rabbit (1:500, A21207, Invitrogen) for 6–8 h at 4°C. If necessary, the sections were incubated with tertiary antisera for 2–4 h in 0.01 m PBS with 0.3% (v/v) Triton X-100 at 4°C. After the immunofluorescence histochemical staining, the sections were observed and images were captured using VS200 microscope (VS200, Olympus, Japan). Digital images were captured using VS200 software (Olympus).

### 2.8. *In vivo* single-unit recording

*In vivo* single-unit recordings were performed as described in previous studies with minor modifications (Hua et al., 2018; Huang et al., 2020). The four guide tubes contained 16-channel electrodes using 25.4-μm formvar-insulated nichrome wire (Cat No. 761500, A-M System, United States) and a 62.5-μm diameter optical multimode fiber in the center. The electrodes implantation was performed at the following stereotaxic coordinates: IC, 1.10 mm anterior to the bregma, 3.0 mm lateral to the midline and 3.75 mm vertical to the skull surface in Sham/SNI mice. Head-fixation was utilized in our study: immediately after the implantation of electrode in the IC, dental adhesive resin cement (Super-bond C&B, Japan) was used to stick the metal head bar to the exposed skull, to ensure the security of the head-fixed position when the head bar was held firmly by the behavioral apparatus during recording (Guo et al., 2014). The mice were given at least 1 week to recover after the implantation of the electrodes. Before recording, mice were habituated to the head-fixed setup for at least 4 sessions (15 min per session, twice/day). Single unit recordings were performed with a Neurostudio System (Jiangsu Brain Medical Technology Co. ltd, China), PACs (15 μL, 100 μM) was administrated into the both nostrils (intranasal administration) between the 20 min Pre-PACs recording period and 30-min post-PACs recording period (Erdo et al., 2018). The electrodes were gradually lowered (~40 μm after each recording day) during 7-day recording period. During recording, the single unit signal was acquired at 30 kHz, band-pass filtered between 300 and 6,000 Hz, and LFPs were band-pass filtered in the range of 1–1,000 Hz. For spike detection, the signal amplitude threshold was set at 50 μV.

### 2.9. Spike sorting and cell type identification

Single-unit spike sorting was performed by MClust-v4.4 toolbox with MATLAB software (MathWorks, United States). To be precise, based on amplitude and waveform energy features, spikes were manually sorted into clusters. A cluster of waveforms was considered as a single neuron if the ratio of its inter-spike intervals (ISI) under 2 ms was <1%, and the unit quality was quantified by isolation distance (>20) and L-ratio (<0.1) (Hua et al., 2018). Besides, the two units were considered as a single neuron when the spike time of all the units measured coincided via the cross-correlation comparison. Pyramidal neurons and gamma-aminobutyric acid (GABA) neurons are two main cell types in IC region. The single neuron was classified as putative pyramidal neuron based on the criteria as described in a previous study (Apkarian et al., 2005): trough-to-peak duration above 430 μs which exhibited long duration action potentials. GABA neurons were identified based on criteria that trough-to-peak duration under 430 μs.

### 2.10. Statistical analysis

Statistical analysis was performed using the GraphPad Prism (GraphPad Software, San Diego, CA, United States). For the paw withdrawal threshold, heat pain, and dynamic mechanical allodynia assessment, data were subjected to unpaired *t*-test. For SNI-induced anxiety-like behaviors, data were subjected to unpaired-*t*-test. For multi-channel recording, the *p* values were calculated by the Wilcox rank-sum test, two-tailed paired and unpaired *t* tests. Results are expressed as mean ± SEM. Statistical significance was set at *p < 0.05; **p < 0.01; ***p < 0.001, ****p < 0.0001.

## 3. Results

### 3.1. Application of PACs induces analgesic effect in SNI mice

To assess the analgesic effects of PACs, ACSF (0.5 μL) or PACs (100 μM, 0.5 μL) was injected into the IC via the implanted cannula in both sham and SNI mice at Day 1 after surgery. Paw withdrawal mechanical thresholds (PWMT) in *von frey* filament test, paw withdrawal thermal latency (PWTL) in Hargreaves test and spontaneous allodynia score by stroking the hindpaw plantar surface with a soft paintbrush were measured to evaluate the possible static and dynamic pain responses of mice (Figure 1A). As shown in Figure 1B, PACs application significantly increased PWMT when compared with ACSF administration in sham group (*p* < 0.05) and SNI group (*p* < 0.0001). Microinjection of PACs in IC had no obvious effect on heat hyperalgesia as PWTL was not significant changed neither in sham nor in SNI groups (Figure 1C). After PACs application, allodynia score was significantly reduced in SNI group (*p* < 0.05, PACs injection vs. ACSF injection) but not significant changed in sham group (Figure 1D). These pain behavioral tests together indicate that SNI surgery induced obvious mechanical allodynia, heat hyperalgesia and spontaneous pain in mice. PACs injection into the IC alleviated mechanical allodynia and spontaneous pain.

### 3.2. Application of PACs induces anxiolytic effects in SNI mice

**Figure 2:**
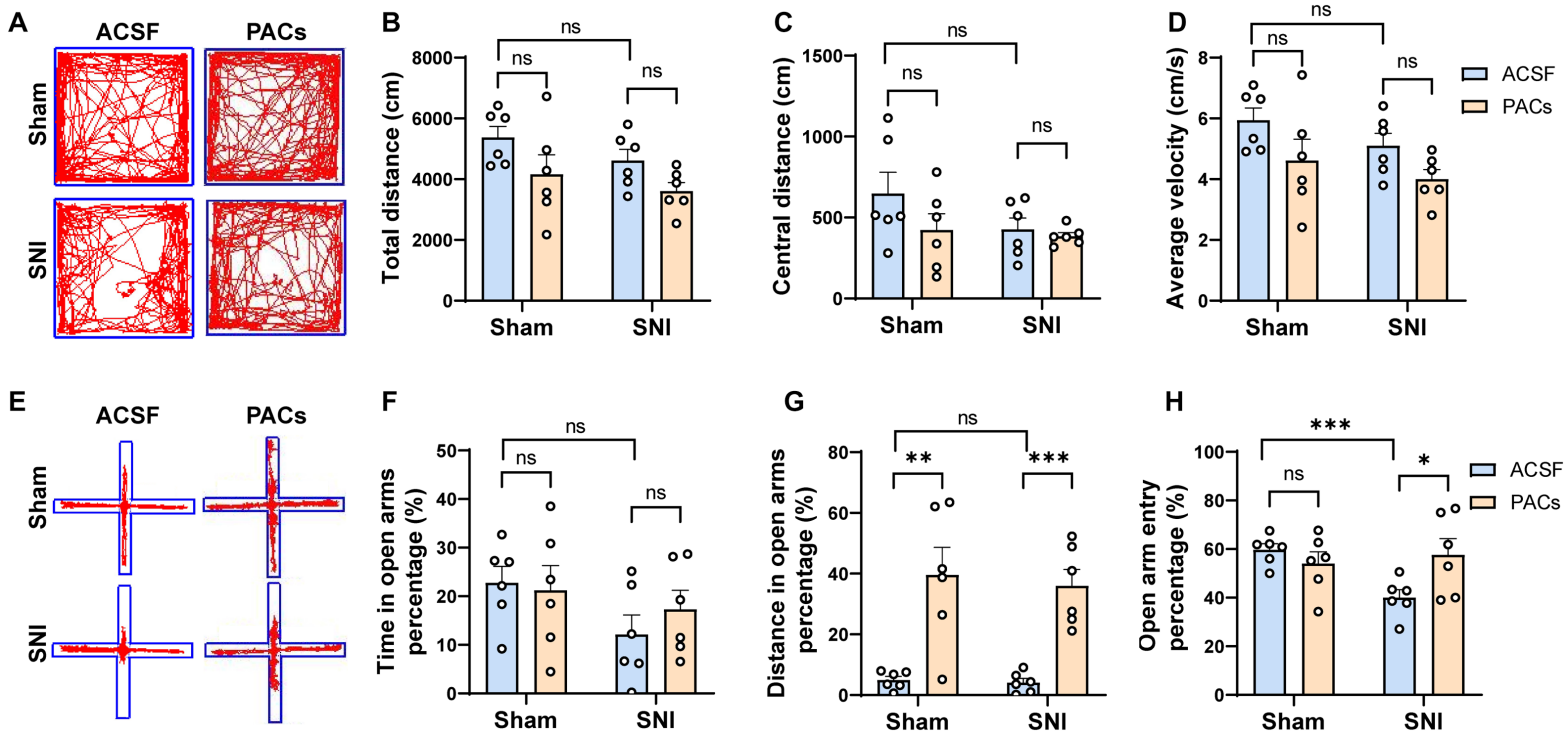
PACs relief the anxiety-like behaviors in SNI mice. **(A)** Representative traces in the OFT test of different groups after PACs or ASCF administration. **(B)** Total distance in sham (*p* = 0.2094, *t* = 1.342, *n* = 6 mice, unpaired *t*-test) and SNI group (*p* = 0.0556, *t* = 2.166, *n* = 6 mice, unpaired *t*-test). SNI vs. sham, *p* = 0.6202, *t* = 0.5222. **(C)** The values of central distance in sham (*p* = 0.2057, *t* = 1.353, *n* = 6 mice, unpaired *t*-test) and SNI group (*p* = 0.5848, *t* = 0.5646, *n* = 6 mice, unpaired *t*-test). SNI vs. sham, *p* = 0.1721, *t* = 1.471. **(D)** The values of average velocity in sham (*p* = 0.1326, *t* = 1.637, *n* = 6 mice, unpaired *t*-test) and SNI group (*p* = 0.0580, *t* = 2.140, *n* = 6 mice, unpaired *t*-test). SNI vs. sham, *p* = 0.1718, *t* = 1.472. **(E)** Representative traces in the EPM test of different groups after PACs or ASCF administration. **(F)** Time in the open arms percentage (%) in sham (*p* = 0.8019, *t* = 0.2576, *n* = 6 mice, unpaired *t*-test) and SNI groups (*p* = 0.3773, *t* = 0.9238, *n* = 6 mice, unpaired *t*-test). SNI vs. sham, *p* = 0.0692, *t* = 2.035. **(G)** Distance in open arms percentage (%) in sham (*p* = 0.0034, *t* = 3.807, *n* = 6 mice, unpaired *t*-test) and SNI group. (*p* = 0.0002, *t* = 5.744, *n* = 6 mice, unpaired *t*-test). SNI vs. sham, *p* = 0.6523, *t* = 0.4644. **(H)** Open arm entry percentage (%) in sham (*p* = 0.3188, *t* = 1.049, *n* = 6 mice, unpaired *t*-test) and SNI group (*p* = 0.0406, *t* = 2.2350, *n* = 6 mice, unpaired *t*-test). SNI vs. sham, *p* = 0.0006, *t* = 4.891. *n.s*., not significant, *p < 0.05; **p < 0.01; ***p < 0.001.

To test whether microinjection of PACs into the IC affects anxiety-like behavior in SNI mice, open field test (OFT) and elevated plus maze (EPM) tests were carried out at day 1 after surgery (Figure 1A). In the OFT experiment, PACs administration did not, although with a tendency, change the total distance, central distance and average velocity in either sham or SNI group (Figures 2A–D), suggesting that PACs have no significant influence in the locomotion behaviors in the open filed test. EPM test was then conducted to check the effects of PACs application on anxiety-like behaviors (Figures 2E–H). Compared with ACSF, PACs administration significantly increased the percentage of distance in the open arms in sham (*p* < 0.001) and SNI group (*p* < 0.001) (Figure 2G). Besides, the percentage of open arm entries was higher after PACs administration in SNI mice (*p* < 0.05, Figure 2H). In together, these results suggest that PACs should relief the anxiety-like behaviors in SNI mice.

### 3.3. PACs reduced FOS expression in pyramidal cells in SNI mice

**Figure 3:**
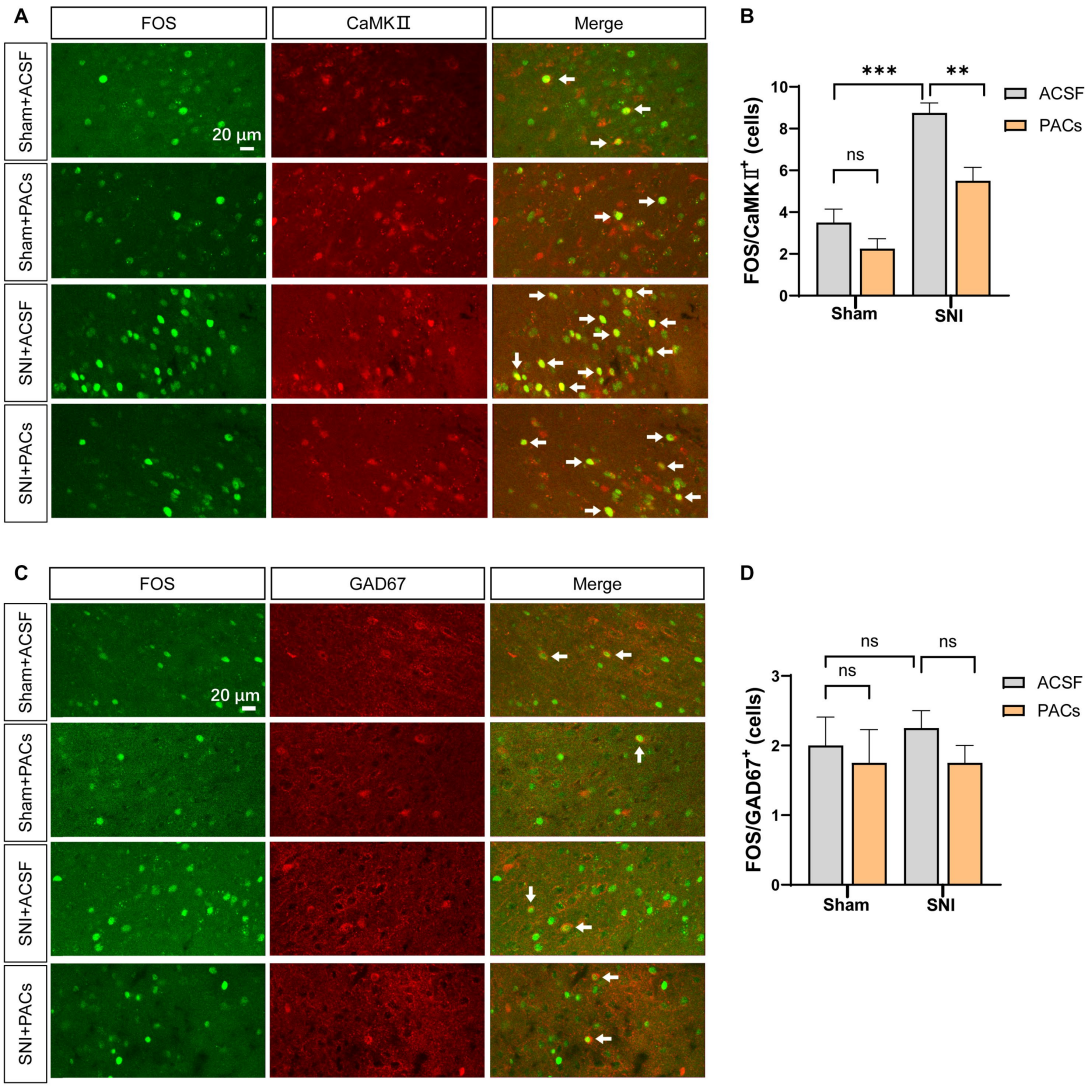
The decreased FOS expression in pyramidal cells but not interneurons in the IC of SNI mice. **(A)** Representative immunofluorescent photos of FOS-immunoreactive (green) and CaMKII-immunoreactive (red) neurons in the IC of sham and SNI mice after intranasal administration of ACSF or PACs. **(B)** The average number of FOS/CaMKII double-labeling neurons in sham (*p* = 0.1708, *t* = 1.555, *n* = 4 mice, unpaired *t*-test) and SNI group (*p* = 0.0068, *t* = 4.044, *n* = 4 mice, unpaired *t*-test). SNI vs. sham, *p* = 0.0006, *t* = 6.533. **(C)** Representative immunofluorescent photos of FOS-immunoreactive (green) and GAD67-immunoreactive (red) neurons in the IC of sham and SNI mice after intranasal administration of ACSF or PACs. **(D)** The average number of FOS/GAD67 double-labeling neurons in sham (*p* = 0.7049, *t* = 0.3974, *n* = 4 mice, unpaired *t*-test) and SNI group (*p* = 0.2070, *t* = 1.414, *n* = 4 mice, unpaired *t*-test). SNI vs. sham, *p* = 0.6202, *t* = 0.5222. *n.s.*, not significant; **p < 0.01; ***p < 0.001.

It is well documented that IC play a pivotal role in the regulation of pain and pain-related anxiety behaviors (Zhuo, 2019), we thus examined whether the activities of IC pyramidal cells and interneurons were changed in SNI mice and affected after intranasal application with PACs. The expression of FOS protein (an activity-dependent neuronal marker) was then explored in the IC by using double-immunofluorescent staining of FOS/CaMKII and FOS/GAD67. The number of FOS/ CaMKII double-labeled (FOS/CaMKII^+^) neurons was significantly increased in SNI + ACSF group in comparison with that in the sham+ACSF group, and the number of FOS/CaMKII^+^ neurons decreased in SNI + PACs group than that in the SNI + ACSF group (Figures 3A,B). Meanwhile, the number of FOS/GAD67^+^ neurons showed no significant changes with the multiple comparisons of each group (Figures 3C,D). The results suggest that the activities of pyramidal cells but not interneurons are enhanced in the IC in mice with neuropathic pain, which could be reduced by intranasal administration of PACs.

### 3.4. PACs suppressed the increased excitability of pyramidal cells in the IC in SNI mice

**Figure 4:**
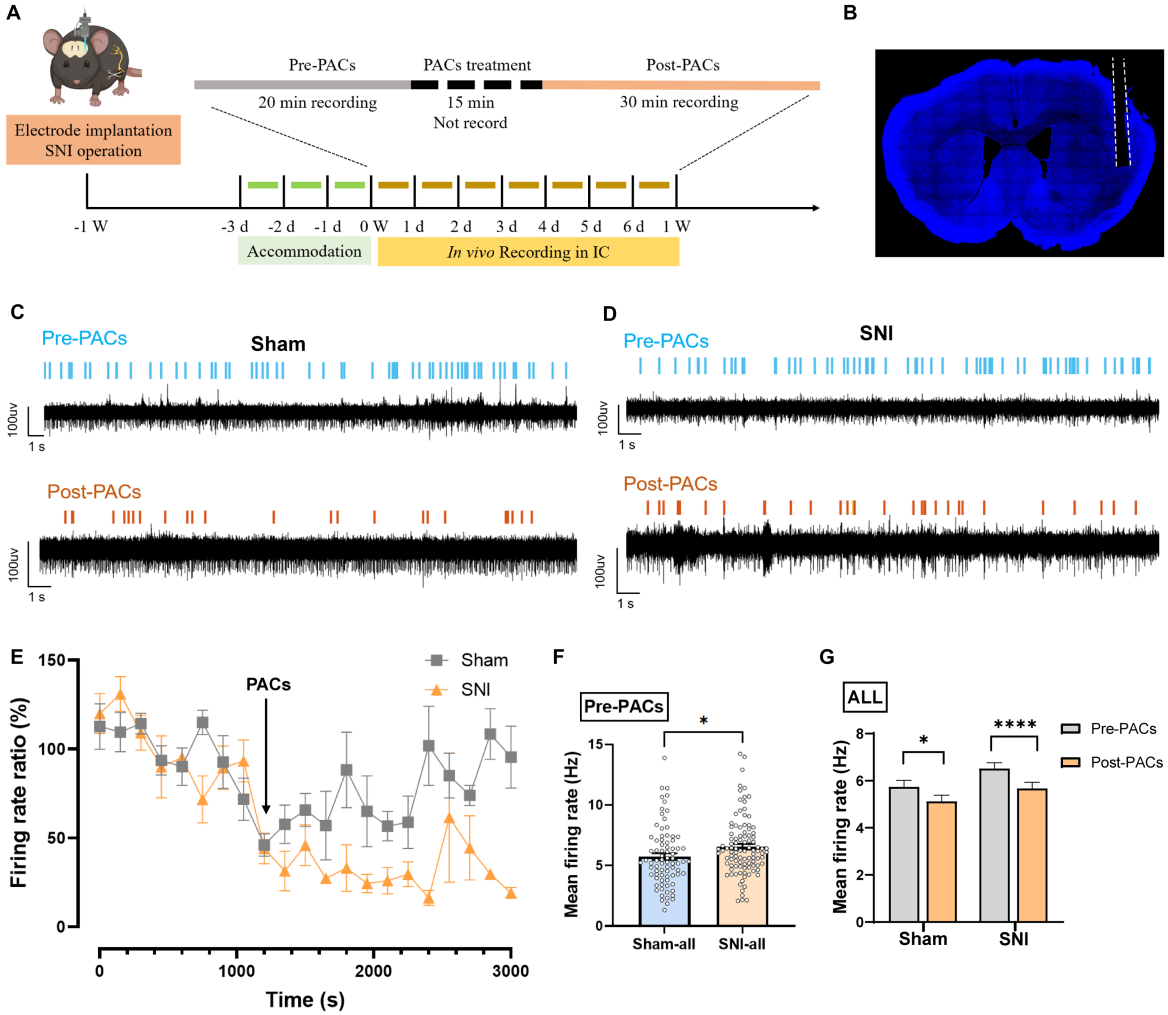
PACs administration decreases the firing rate of IC neurons. **(A)** *In vivo* multi-channel recording timeline. **(B)** Representative image of electrode implantation trace in the IC. **(C,D)** Example recording signals of IC neurons. The blue or orange lines indicate the spikes in one sham-operated or one SNI mouse, respectively. **(E)** The percentage of firing rate of IC neurons in pre- and post-PACs sessions (*n* = 3 channels in 3 mice). **(F)** The mean firing rate of all recorded neurons in pre-PACs session in sham (*n* = 79 neurons) and SNI groups (*n* = 102 neurons) (*p* = 0.0260, *t* = 2.245, *n* = 6 mice in each group, unpaired *t*-test). **(G)** The PACs administration decreases the mean firing rate of all recorded neurons in sham group (*p* = 0.0107, *t* = 2.616, *n* = 6 mice, paired *t*-test) and SNI group (*p* < 0.0001, *t* = 4.720, *n* = 6 mice, paired *t*-test). *n.s.*, not significant; *p < 0.05; ****p < 0.0001.

To evaluate whether PACs directly affect the *in vivo* activities of pyramidal cells and interneurons in the IC, we implanted a 16-channel electrode into the IC for single-unit recording of the cellular spiking (Figures 4A,B). PACs were intranasal administrated, while 20-min baseline spike recording (pre-PACs) and 30-min post-PACs spike recording was performed (Figures 4C–E). We found that the mean spike frequency of well-isolated neurons in the baseline was significantly increased in SNI group when compared with that in sham group (*p* < 0.05) (Figure 4F), and PACs administration decreased the mean firing rate of all recorded neurons in both sham (*p* = 0.0107) and SNI groups (*p* < 0.0001) (Figure 4G).

**Figure 5:**
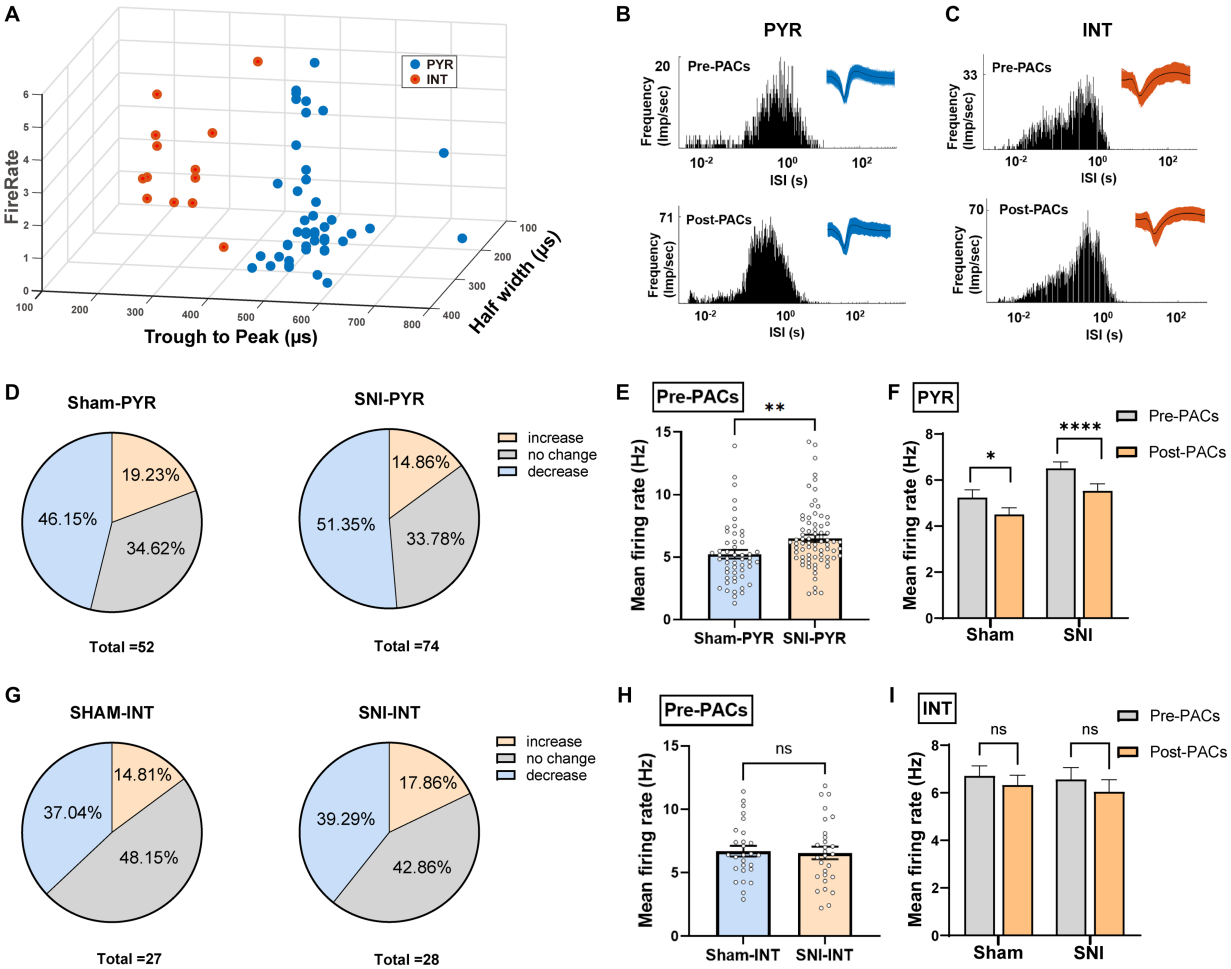
PACs administration decreases the activities of pyramidal cells not interneurons in the IC. **(A)** IC neurons are classified into pyramidal cells and interneurons using k-means cluster-separation algorithm based on their electrophysiological properties. **(B,C)** Histograms of the inter-spike intervals (ISI) from the spikes of a pyramidal cell and an interneuron in pre- and post-PACs sessions. Insets at the top right corner show the waveforms of the detected single unit. **(D)** Proportion of pyramidal cells with changed firing rate in sham and SNI groups. Pie charts summarize the changes in firing rate of pyramidal cells in sham (*n* = 52 neurons) or SNI (*n* = 74 neurons) groups. Pre- vs. post-PACs administration, Wilcoxon rank-sum test. **(E)** The mean firing rate of pyramidal cells in sham (*n* = 52 neurons) and SNI group (*n* = 74 neurons) during pre-PACs session (*p* = 0.0054, *t* = 2.830, *n* = 6 mice, unpaired *t*-test). **(F)** The mean firing rate of the pyramidal cells in the SNI group (*p* < 0.0001, *t* = 4.424, *n* = 74 neurons of 6 mice, paired *t*-test) and sham group (*p* = 0.0209, *t* = 2.384, *n* = 52 neurons of 6 mice, paired *t*-test) during the Pre-PACs and post-PACs period. **(G)** Proportion of interneurons with changed firing rate in sham and SNI groups. Pie charts summarize the changes in firing rate of interneurons in sham (*n* = 27 neurons) or SNI (*n* = 28 neurons) groups. Pre- vs. post-PACs administration, Wilcoxon rank-sum test. **(H)** The mean firing rate of interneurons in sham (*n* = 27 neurons) and SNI group (*n* = 28 neurons) during pre-PACs session. (*p* = 0.8229, *t* = 0.2249, *n* = 6 mice, unpaired *t*-test). **(I)** The mean firing rate of the interneurons in sham (*p* = 0.2835, *t* = 1.095, *n* = 27 neurons of 6 mice, paired *t-*test) and SNI group (*p* = 0.0938, *t* = 1.737, *n* = 28 neurons of 6 mice, paired *t-*test) after PACs administration. *n.s.*, not significant; *p < 0.05; **p < 0.01; ****p < 0.0001.

To further test whether the PACs administration affected the activity of pyramidal cells and interneurons in the IC, the two types of neurons were then classified based on their trough-to-peak duration, firing rate and half width as described in our previous study (Figure 5A; Zhu et al., 2022). The waveform characteristics and the internal-spiking interval (ISI) of each isolated neuron were assessed to ensure the two units isolated in the pre-PACs and the post-PACs sessions were from the same neuron (Figures 5B,C). In sham operated mice, PACs increased the firing rate of 19.23% of pyramidal cells and decreased the firing rate of 46.15% of pyramidal cells, while PACs increased the firing rate of 14.86% of pyramidal cells and decreased the firing rate of 51.35% pyramidal cells in SNI mice (Figure 5D). The enhanced firing rate of the pyramidal cells in the SNI group (*p* < 0.01) (Figure 5E) was significantly reduced after PACs administration (*p* < 0.0001) (Figure 5F). Meanwhile, intranasal administration of PACs increased 14.81% and decreased 37.04% of the interneurons isolated in sham operated mice, and increased 17.86% and decreased 39.29% of interneurons in SNI group (Figure 5G). The mean firing rate of the interneurons in the two groups showed no significant different (*p =* 0.8229) (Figure 5H) and PACs administration did not affect the mean firing rate of the interneurons in either sham (*p =* 0.2835) or SNI mice (*p =* 0.0938) (Figure 5I). These results indicate that only the activities of pyramidal cells but not interneurons in the IC are decreased by PACs administration. Meanwhile, PACs have stronger inhibitory effect on the pyramidal cells in SNI group in comparison with those in the sham group.

## 4. Discussion

In this study, we verified the analgesic effect and anxiolytic effect of PACs in mice with neuropathic pain at cortical level. We further found that the increased activity of pyramidal cells in the IC subjected to painful responses and pain-related anxiety. The analgesic and anxiolytic effect of PACs administration is closely involved with their inhibition of the activity of pyramidal cells in the IC.

PACs is widely known for their antioxidant, antiapoptotic and antiallergic effects, which are usually administrated orally and absorbed from the gastrointestinal tract (Del Rio et al., 2010; Martinez-Micaelo et al., 2015). A previous study showed that oral gavage of 90 mg/kg PACs in mice is the optimal does for the attenuation of neuropathic pain via suppressing matrix metalloproteinase-9/2 (Pan et al., 2018). Our previous study pointed out intrathecal injection of 20 μg PACs induced obvious anti-inflammatory pain effect at the spinal cord level by inhibiting phosphorylated activation of the PI3K pathway (Fan et al., 2021). Our present study aimed to observe the PACs’ effects at cortical level. PACs were injected into the IC through a cannula in the behavioral tests. However, in morphological experiment and *in vivo* electrophysiological recording, injection may affect the local brain structures and ion concentration around the cannula or electrodes and PACs was thus applied through intranasal delivery. PACs administration showed obvious alleviation of mechanical allodynia and spontaneous pain, but not of heat hyperalgesia. However, in our previous works, we have shown that intrathecal injection of PACs inhibits both mechanical and heat pain responses (Fan et al., 2021). These different analgesic effects on heat hyperalgesia suggest that PACs may induce different analgesic effect in spinal and cortical level. The anxiolytic effects of *p.o.* application of PACs have been proved in different animal disease models (Subash et al., 2016; Jiang et al., 2017), our present study confirms that the PACs could directly affect the cortical activity and induce anxiolytic effect without through the Gut-brain-axis.

In cortex pyramidal cells (70–80% of the total number of neurons) release glutamate and exert excitatory roles while interneurons (20–30% of the total number of neurons) release GABA and play inhibitory effects (Tremblay et al., 2016; Yavorska and Wehr, 2016). Our previous work points out that the *in vivo* firing of pyramidal cells increases and interneurons stay unchanged in the anterior cingulate cortex after neuropathic pain (Zhu et al., 2022) which is in consistent with our present results in the IC. Therefore the unbalanced excitatory and inhibitory ratio of cortical neurons might be important for the occurrence and process of chronic pain and related negative emotion. Inhibition of the enhanced spiking of pyramidal cells but not interneurons may potent enough for analgesic and anxiolytic effects. However considering the important roles of cortical interneurons in the regulation of pain and anxiety (Kang et al., 2015; Shao et al., 2021) further works involving in silence of cortical pyramidal cells and excitation of interneurons should be carried out to validate this hypothesis in the future experiments.

In conclusion, we prove that PACs induce analgesic and anxiolytic effects by inhibiting the enhanced spiking of cortical pyramidal cells in mice with neuropathic pain, which should provide new methods for clinical treatment of chronic pain and anxiety comorbidity.

## Data availability statement

The original contributions presented in the study are included in the article/supplementary material, further inquiries can be directed to the corresponding authors.

## Ethics statement

The animal study was reviewed and approved by the Animal Care Committee of the Fourth Military Medical University.

## Author contributions

TC, M-MZ, and PW conceived the project and designed the experiments. PW, H-XS, and HZ performed the *in vivo* recording study. H-XS, X-DL, and JW completed morphological staining. D-YZ, K-KX, and T-TC performed the behavioral tests. TC and PW drafted the manuscript. M-MZ helped to finish the final version of the manuscript. All authors have read and approved the final manuscript.

## Funding

This work was supported by the National Natural Science Foundation of China (32192410 and 32071000 to TC) and Scientific and Technological Project of FMMU (2021HKYX06 to M-MZ).

## Conflict of interest

The authors declare that the research was conducted in the absence of any commercial or financial relationships that could be construed as a potential conflict of interest.

## Publisher’s note

All claims expressed in this article are solely those of the authors and do not necessarily represent those of their affiliated organizations, or those of the publisher, the editors and the reviewers. Any product that may be evaluated in this article, or claim that may be made by its manufacturer, is not guaranteed or endorsed by the publisher.
